# Anterior knee pain as a potential risk factor for falls in older adults: insights from the osteoarthritis initiative data

**DOI:** 10.1186/s12889-023-17237-8

**Published:** 2023-11-20

**Authors:** Ting Xiong, Yanghuan Ou, Shenliang Chen, Shuaigang Liu, Xuan Yi, Xueqiang Deng, Tao Cheng, Liang Hao

**Affiliations:** 1 Department of Orthopedics, Second Affiliated Hospital of Nanchang University, 1 Minde Road, Nanchang, Jiangxi Province 330006 China; 2 Department of Orthopedic Surgery, Shanghai Sixth People’s Hospital, Shanghai Jiao Tong University School of Medicine, Shanghai, China

**Keywords:** Accidental falls, Older adults, Osteoarthritis, Pain, Knee

## Abstract

**Background:**

Knee joint pain has been demonstrated to be a separate risk factor for falling. A common pain site in the knee, anterior knee pain(AKP), is believed to be associated with early knee osteoarthritis (KOA).This study investigated the relationship between falls and AKP in people with or at risk for KOA.

**Methods:**

Four years of follow-up data from the Osteoarthritis Initiative cohort trial, a large-scale, multicenter observational investigation, were analyzed in this study. A patellar quadriceps tenderness/tendinitis knee exam was performed to evaluate AKP. Falls were self-reported. The associations between falls (recurrent falls: ≥2 falls/year; any falls: ≥1 fall(s)/year) and AKP were analyzed using the generalized estimation equation of repeated logistic regression and adjusted for confounding variables.

**Results:**

The study analyzed data from 3,318 participants, split into two groups: those with AKP (720 participants) and those without AKP (2,598 participants). The primary outcome of the study, which focused on repeated falls, revealed that participants with AKP were 1.27 times more likely to experience repeated falls compared to those without AKP (95% CI: 1.07–1.52, *P* = 0.007). However, when considering any falls experienced by an individual as an additional outcome, it is important to note that our findings did not indicate a significant predictive effect of AKP on any falls investigated. Sensitivity analyses, which excluded knee arthroplasty cases, yielded consistent results with the aforementioned findings.

**Conclusions:**

Older adults with AKP experience a higher frequency of falls compared to those without AKP in individuals diagnosed with KOA or at a high risk of developing KOA.

**Supplementary Information:**

The online version contains supplementary material available at 10.1186/s12889-023-17237-8.

## Introduction

Falls and associated injuries are prevalent among middle-aged and older adults. Falls not only cause immediate harm but also have long-term consequences. Approximately one in five falls results in serious injuries, such as fractures or head injuries [1]. Each year, more than 800,000 patients are hospitalized due to fall-related injuries, with head injuries and hip fractures being the most frequent [2]. Falls can lead to functional decline, an increased need for long-term care, and heightened mortality among middle-aged and older adults [3, 4]. Data from the 2014 Behavioral Risk Factors Monitoring System Survey, studied by the Centers for Disease Control and Prevention in the United States, indicates that more than 25% of elderly respondents reported falling, with fatalities from falls reaching approximately 27,000 among older individuals [5, 6].

Falls can be attributed to various factors, one of which is pain. Pain independently contributes to the risk of falls. Patients who report pain at multiple sites have a higher risk of falling than those who report pain at one site [7]–9]. Pain is also a primary symptom of osteoarthritis (OA) [10]. In the United States, lower limb pain from OA is a main factor leading to activity disturbances in middle-aged and older adults [10, 11]. Previous studies have demonstrated an association between knee joint pain and falls. The relationship is often assessed using overall knee pain in most studies. However, there is a generally accepted association between structural damage of the knee joint, especially the patellofemoral joint, and the occurrence of anterior knee pain (AKP). Whereas we used AKP in this study to assess knee pain in patients, which is a common cause for seeking medical attention. AKP typically includes patellofemoral pain and affects 2.5 million runners per year, of which 70% decreased their overall physical activity levels at least five years after their first injury [12]. Previous studies investigating the progression of knee OA and its association with knee joint pain have yielded controversial findings. Patient-reported pain does not consistently align with the progression of knee OA. In contrast, recent research has discovered a significant association between AKP and the occurrence of structural changes in the patellofemoral joint among patients with patellofemoral OA. Patellofemoral OA typically manifests at an early stage of knee OA, occurring before OA progresses to the tibiofemoral joint [13]–15]. The study findings revealed that the presence of AKP is indicative of knee OA progression. This extends the existing research on the association between AKP and knee OA. Thus, we aimed to investigate whether AKP is also associated with an elevated risk of falls in patients.

There is a lack of reported studies on the association between AKP and falls. This study establishes a relationship between the presence of AKP at baseline and the occurrence of recurrent falls (two or more falls) and any falls (one or more falls) among patients with knee OA or those at risk of developing it. Our hypothesis is that individuals experiencing AKP have a higher susceptibility to falls when compared to those without AKP. This study aims to offer clinical practitioners novel insights into the physical condition and disease progression of patients at a high risk of knee OA and AKP.

## Methods

### Osteoarthritis initiative

The Osteoarthritis Initiative (OAI) cohort study was an extensive multicenter observational study with a primary focus on knee OA. Participants aged 45–79 years at five clinical sites (John Hopkins Bayview Medical Center and the University of Maryland, Baltimore, Maryland; Ohio State University, Columbus, Ohio; University of Pittsburgh, Pittsburgh, Pennsylvania; and Memorial Hospital, Pawtucket, Rhode Island) who participated in the OAI between 2004 and 2006 were included in this study. The two major sub cohorts of the OAI included participants with symptomatic knee OA at baseline who were monitored for progression of OA (the progression sub cohort) and participants who did not have any symptoms of knee OA but had certain traits that increased the risk of developing symptomatic knee OA during the study period (the incidence sub cohort). The progression sub cohort was defined as participants with symptomatic tibiofemoral knee OA at baseline, frequent knee symptoms, and radiographic tibiofemoral knee OA diagnosed in at least one native knee at baseline. The incidence sub cohort was defined as participants who had no symptomatic knee OA in either knee at baseline but had other characteristics, such as knee symptoms in a native knee within the previous 12 months, overweight/obesity, knee injury or surgery, family history, Heberden’s nodes, repetitive knee bending, and an age of 70–79 years. Participants with rheumatoid arthritis, inflammatory arthritis, or pregnancy were excluded from the study. Participants who were unlikely to reside near the clinic for at least 3 years were also excluded intended to ensure that participants could be followed up adequately during the study period. The OAI cohort study included a small control group consisting of individuals who did not have the risk factors required for eligibility at baseline, did not experience any symptoms related to the knee joints, and had no imaging evidence of knee OA. As it focused on patients with knee OA and those at high-risk for OA, a control group was not included. The OAI cohort study was approved by the institutional review boards at the participating OAI facilities. All participants provided written informed consent for their participation in this study. The OAI cohort study was conducted according to the principles of the Declaration of Helsinki. As patients’ falls at year five were not documented in the OAI database, we retrospectively collected data from a four-year follow-up period spanning from baseline to year four as the main focus of current study. These data and additional details are publicly available at https://nda.nih.gov/oai/.

### Independent variable: AKP

Each participant underwent a standardized physical examination of the knee during the enrolment visit. The physicians and clinical personnel at each participating institution underwent thorough training, and the knee examinations were performed by qualified personnel at each location under observation by medical examiners. The patellar quadriceps tenderness/tendinitis knee examination was used to evaluate AKP [16, 17]. The examination began with the participant seated with their knees flexed at 90º. The clinic personnel used their thumbs to examine for tenderness in four different locations around the patella: the bony attachment of the quadriceps tendon at the patella; the distal end of the quadriceps muscle as it forms the quadriceps tendon (quad muscle/tendon transition); the bony attachment of the infrapatellar tendon at the patella; and below the patella (the thumb and forefinger were squeezed to apply pressure inferior to the infrapatellar tendon). The researcher evaluated the lower extremities of all patients included in the study. In order to determine whether a patient can be categorized as not having AKP in this study, there should be no tenderness detected in any of the four above locations in both legs. Conversely, if tenderness is observed in any of the four above locations in both legs, the patient is considered afflicted with AKP.

### Dependent variable: falls

The participants self-reported the number of times they had fallen and landed on floor or ground in the previous year. The data were recorded as none, one, two, three, four, five, or six or more falls. In this study, the primary outcome measure was recurrent falls, while the secondary outcome measure was any falls, as previously described [18]. In this study, participants who experienced two or more falls within the past year were classified as having recurrent falls, while those who reported at least one fall within the past year were classified as having any fall. Data on falls were gathered annually throughout the four-year follow-up period of the study.

### Covariates

Major determinants of fall risk were identified from systematic reviews regarding data of middle-aged and older individuals living in communities [19]. The following variables were assessed at the baseline visit: age, sex, race, body mass index (BMI), education, smoking history, cohort (the progression sub-cohort or the incidence sub-cohort), alcohol use, analgesics use, Charlson Comorbidity Index (CCI) [20], Center for Epidemiologic Studies Depression (CES-D) Score [21], Physical Activity Scale for the Older adults (PASE) score [22], repeated chair stands [23, 24], pace, knee confidence, and history of falls (at baseline). Race was divided into white and non-white based on the medical records [25]. BMI was categorized as normal or obese [26]. Education was divided into post-college and non-post-college groups. The CCI was defined as a three-category variable based on previous research [27]. The participants were divided based on their ability to complete at least five repeated chair stands. The 20-meter walk test was used to assess the participants’ pace [28]. The knee confidence was self-reported using the Knee Injury Osteoarthritis Outcome Score question, “How much are you troubled with lack of confidence in your knees?” [29]. The participants’ fall histories were recorded based on the response to, “Fallen and landed on floor or ground in the past 12 months” [19].

### Statistical analyses

The participants were divided into two groups according to the presence or absence of AKP. The participants’ baseline characteristics are described as mean and standard deviation (SD) or number and percentage. The generalized estimating equation (GEE) is a statistical method used to estimate the parameters of a generalized linear model that considers the possibility of unknown correlations between outcomes. As a history of falls experienced by participants is not an independent variable, previous falls may have influenced the occurrence of falls during the study period. Repeated logistic regression using GEEs and a binomial distribution was used to investigate the association between AKP and falls. The working correlation structures utilized by the GEE models are interchangeable. To determine whether any synergistic effects existed between the confounders, AKP, and their two-way interaction terms, the analysis was conducted by incorporated these as independent variables in the GEE model. Two models were developed: the unadjusted and multivariate models. Stratified analyses were performed to assess the influence of AKP on fall prediction in diverse populations, taking into account age and gender as variables in a study where recurrent falls served as the outcome. Sensitivity analysis was conducted, excluding patients who underwent knee arthroplasty. All statistical analyses were conducted using SPSS software (version 26.0; IBM SPSS Inc., Armonk, NY, USA). Statistical significance was defined at the level of *P* < 0.05. The GEE model produced both the odds ratio (OR) and a 95% confidence interval (CI).

## Results

**Fig. 1:**
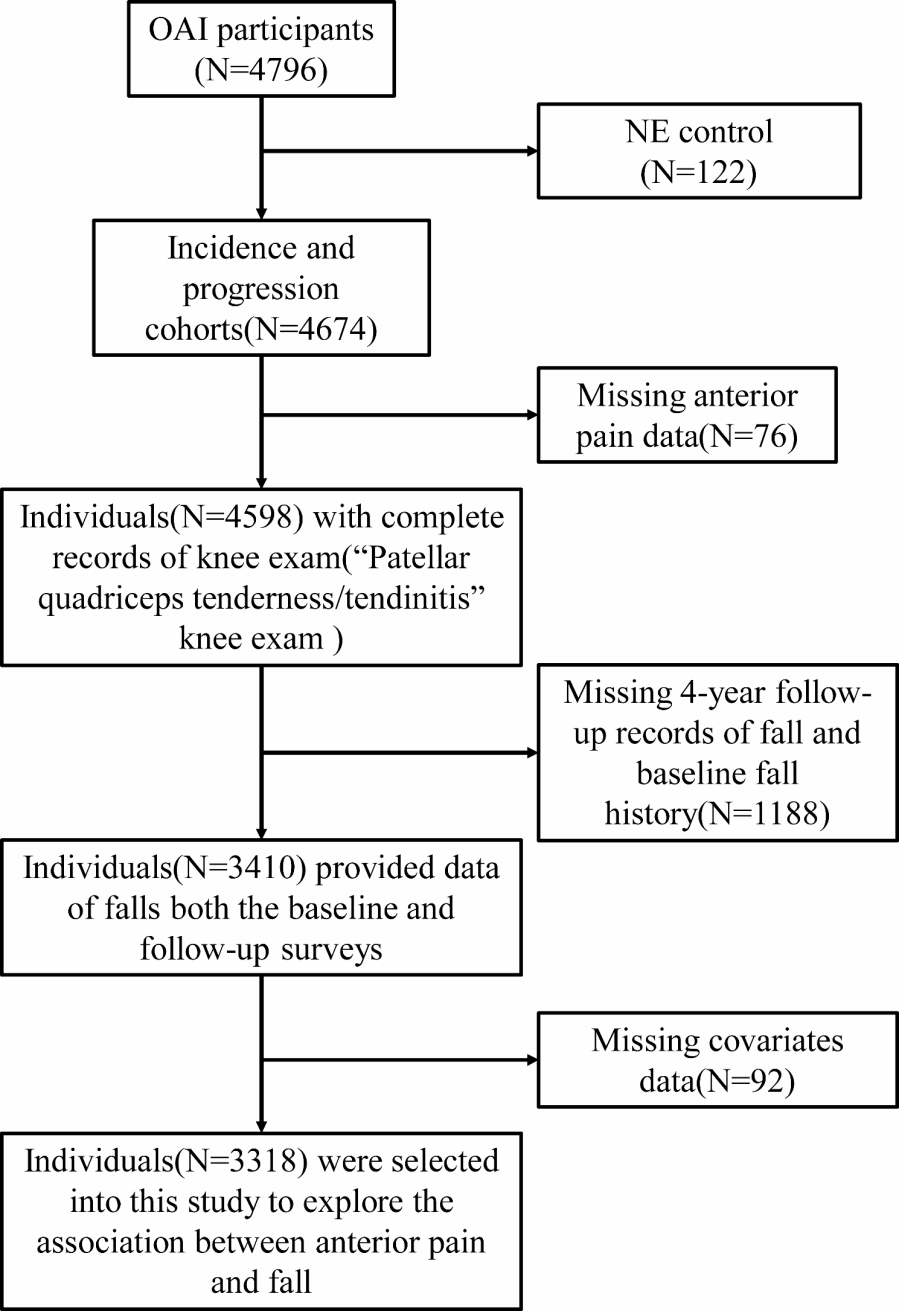
Flow chart of research object screening

We initially excluded the control group cohort as our focus is on individuals affected by knee OA. Moreover, we excluded subjects with missing covariates and those without AKP data. Ultimately, our study included a total of 3,318 participants, with 720 (21.7%) experiencing AKP and 2,598 (78.3%) without AKP constituting the referent group (Table 1; Fig. 1). Baseline characteristics are summarized in Table 1. The AKP group is more likely to comprise of female individuals, and those who are overweight. On the other hand, the reference group is predominantly composed of individuals of White race, who possess higher education levels, engage in higher levels of alcohol consumption, exhibit less progression of knee osteoarthritis, have fewer comorbidities, and report a lower frequency of pain medication use. The evaluation of the population using CSE-D scale demonstrated that the reference group obtained lower depression scores. Furthermore, the 20-meter walking test revealed that the reference group exhibited superior physical strength in comparison to the AKP group. Conversely, the AKP group displayed lower confidence levels and a higher incidence of falls. No significant differences were observed between the two groups in terms of age, smoking history, PASE score, and completion rate of the five repeated chair standing tests.

**Table 1:** The comparison between factors in the AKP and non- AKP groups

Variables	Non- AKP(n = 2598)	AKP(n = 720)	*P*
Age(year), mean, SD	61.4(9.0)	61.7(9.2)	0.49
Female, n (%)	1405(54.1)	529(73.5)	< 0.01
White, n (%)	2248(86.5)	538(74.7)	< 0.01
Post college education, n (%)	1118(43.0)	232(32.2)	< 0.01
BMI^*^, n (%)			
Obesity (≥ 30 kg/m^2^)	897(34.5)	312(43.3)	< 0.01
Cohort			
progression	633(24.4)	281(39.0)	< 0.01
History of smoking, n (%)	1187(45.7)	351(48.8)	0.15
Alcohol use, n (%)	2169(83.5)	555(77.1))	< 0.01
Analgesics use, n (%)	85(3.3)	58(8.1)	< 0.01
Charlson Comorbidity Index			
0	2044(78.7)	498(69.2)	< 0.01
1	341(13.1)	137(19.0)	< 0.01
1^+^	213(8.2)	85(11.8)	< 0.01
CES-D^†^	5.56(6.04)	8.35(7.77)	< 0.01
Pase^‡^	162.9(79.5)	159.4(84.3)	0.30
Repeated chair stands^§^	2487(95.7)	692(96.1)	0.65
Pace^||^(m/s)	1.35(0.20)	1.25(0.21)	< 0.01
Lack of knee confidence^**^	1261(48.5)	507(70.4)	< 0.01
Fallen past year, n (%)	791(30.4)	285(39.6)	< 0.01

Abbreviation: AKP: anterior knee pain

^*^Body Mass Index; ^†^Center for Epidemiologic Studies Depression (CES-D) Score; ^‡^Physical Activity Scale for the Elderly (Pase) score; ^§^Repeated chair stands: able to complete 5 stands;^||^20-meter walk: pace (meter/seconds); ^**^Quality of life: how much troubled with lack of confidence in knee(s)

**Fig. 2:**
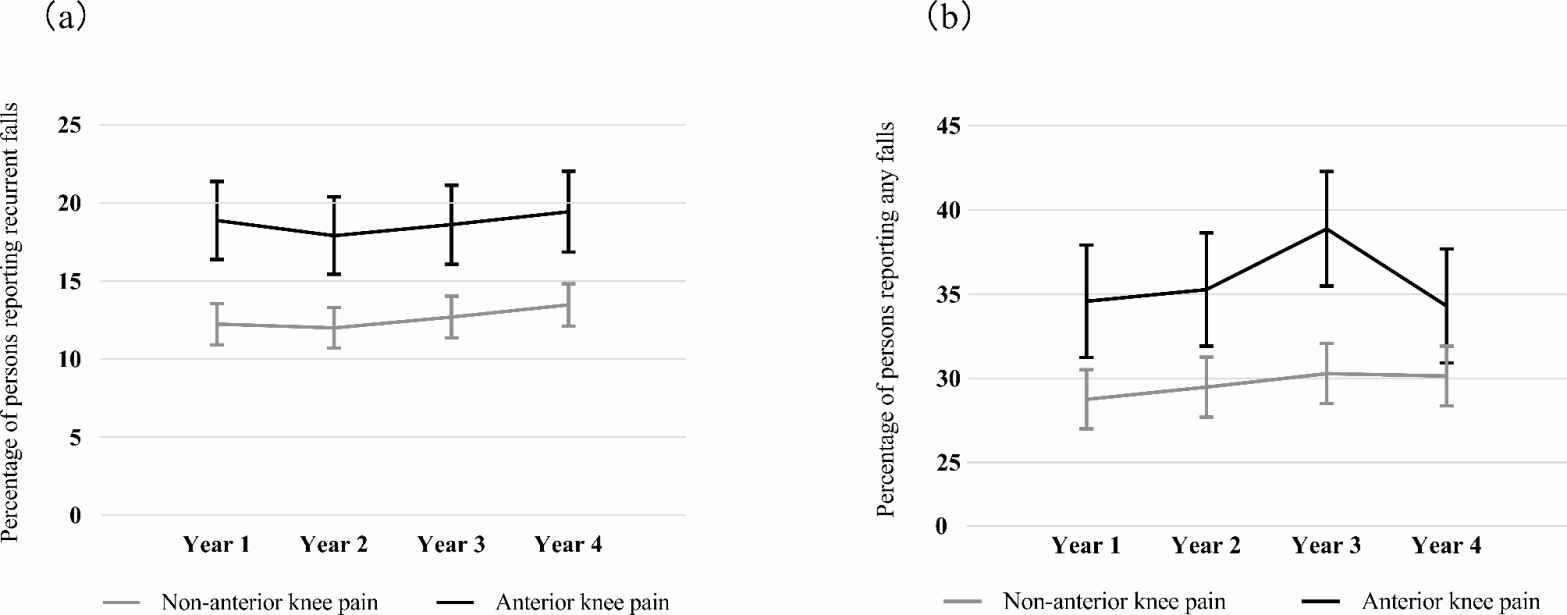
(**a**) Yearly prevalence of recurrent falls rates over a 4-year period for persons. Error bars represent bounds of 95% confidence intervals. (**b**) Yearly prevalence of any falls rates over a 4-year period for persons. Error bars represent bounds of 95% confidence intervals

Throughout the 4-year follow-up period, the AKP group exhibited a notably greater annual rate of falls compared to the non-AKP group, for both the primary and secondary outcomes of the study (Fig. 2). The analysis results, focusing on recurrent falls as the primary outcome, demonstrate that participants with AKP were more likely to experience recurrent falls than those without AKP (OR: 1.60; 95% CI:1.36–1.87, *P* < 0.001). After adjusting for confounders, including age, BMI, gender, race, education, cohort, history of smoking, alcohol use, analgesics use, history of fall, Charlson comorbidity index, CES-D, PASE, Repeated chair stands, pace, and knee confidence, the AKP group was also likely to experience recurrent falls than the non-AKP group (Table 2). However, in the analysis, focusing on any fall as a secondary outcome, indicates an interaction between CES-D and AKP (*P* = 0.031). Therefore, when utilizing any falls as an outcome in the analysis, the participants were stratified by CES-D score (< 16 and ≥ 16) [30]. Among participants with a CES-D score < 16, those with AKP had a 1.32 times higher likelihood of falling than those without AKP (95% CI: 1.16–1.50, *P* < 0.001). Upon adjusting for confounders, participants with AKP still had a 1.16 times higher likelihood of falling than those without AKP (95% CI: 1.02 − 1.33, *P* = 0.029). However, AKP was no significantly associated with falls among patients with a CES-D score ≥ 16 (Table 3).

**Table 2:** Odds Ratio (95% Confidence Intervals) for AKP in the recurrent falls group. ^†^Adjusted covariates: Age, BMI, gender, race, education, cohort, history of smoking, alcohol use, analgesics use, history of fall, Charlson comorbidity index, CES-D, PASE, Repeated chair stands, pace, and knee confidence. note: CES-D × AKP interaction, *P* = 0.084

	Recurrent Falls		
	OR	95%CI	*P*
AKP *(reference of non-AKP)*			
Unadjusted Model	1.60	1.36–1.87	< 0.001
Multivariate Model^†^	1.27	1.07–1.52	0.007

**Table 3:** Odds Ratio (95% Confidence Intervals) for any fall in different depression group

	CES-D < 16			CES-D ≥ 16		
	OR	95%CI	*p*	OR	95%CI	*p*
AKP (reference of non-AKP*)*						
Unadjusted Model	1.32	1.16–1.50	< 0.001	1.05	0.76–1.44	0.782
Multivariate Model	1.16	1.02–1.33	0.029	0.91	0.66–1.27	0.594

^†^Adjusted covariates: Age, BMI, gender, race, education, cohort, history of smoking, alcohol use, analgesics use, history of fall, Charlson comorbidity index, PASE, Repeated chair stands, pace, and knee confidence. note: CES-D × AKP interaction, *P* = 0.031

Participants aged ≥ 65 years with AKP were more likely to experience recurrent falls than those without AKP in the unadjusted (OR: 1.81; 95% CI: 1.38–2.37, *P* < 0.001) and adjusted (OR: 1.40, 95% CI: 1.06–1.87, *P* = 0.019) models. However, this association was not observed in participants < 65 years of age after adjusting for confounders (OR: 1.19; 95% CI: 0.95–1.50, *P* = 0.126) (Supplementary Table 1). Male patients with AKP were more likely to experience recurrent falls than those without AKP in the unadjusted model and multivariate model (OR: 1.90, 95% CI: 1.44–2.50, *P* < 0.001; OR: 1.45, 95% CI: 1.07–1.96, *P* = 0.017 respectively). However, AKP was not significantly associated with recurrent falls among female participants in the multivariate model (OR: 1.17, 95% CI: 0.94–1.45, *P* = 0.161) (Supplementary Table 2). The outcomes were consistent when 350 participants with a history of knee arthroplasty at baseline were excluded from the sensitivity analyses (Supplementary Tables 3, 4).

## Discussion

In this study, we found that AKP is an independent predictor of recurrent falls in individuals with knee OA or those at high risk for it. To investigate the connection between AKP and falls further, we performed an analysis with any falls as the secondary outcome. The findings revealed that the link between AKP and any falls was only significant in patients without depression, whereas this relationship was not evident in individuals with depression. Nonetheless, we should note that the reporting of single falls may have been coincidental [31, 32]. More researchers tend to focus on recurrent falls as the primary outcome of their studies [33, 34]. Furthermore, depression can also elevate the risk of falls among older adults. Consequently, in studies where any individual fall is considered a secondary outcome, the influence of AKP on single falls in patients with depression might be overshadowed by the impact of depression itself on falls.

Nevertheless, our findings align with prior research outcomes [35]. Several reports have explored the association between pain and falls, underscoring an augmented risk of falls in middle-aged and elderly patients afflicted with knee joint pain. Muraki et al. [36] reported that knee pain is independently associated with numerous falls in Japanese women. Additionally, Patel et al. [37] reported that individuals with multiple sites of pain had higher fall prevalence rates than those with pain at a single site. The current study complements the results of previous studies. However, in contrast to previous cross-sectional studies, longitudinal data was analyzed in this study. Furthermore, as opposed to general knee pain, this study focused on pain in specific areas of the knee-AKP, which is a common pain site in the knee patients seeking for treatment, and believed to be associated with early knee OA.

Individuals with AKP may experience quadriceps femoris strength loss and decreased knee joint balance, which are associated with falls and mortality in middle-aged and older adults [38, 39]. Negative effects of AKP may contribute to falls. In addition, AKP in individuals with knee OA and those at high risk for knee OA may indicate concurrent disorders that also induce AKP [40, 41].

Nevertheless, in comparison to healthy individuals, patients with AKP exhibit lower walking speed and frequency, as well as decreased step length and stride. This could be seen as a protective mechanism that can reduce the counteractive force of the quadriceps muscle and thus decrease force on the patella [42].

Hierarchical analysis revealed that AKP was effective in predicting falls in male participants ≥ 65 years of age. The fall risk awareness scores decrease with age [43]. Middle- and older-aged adults who are aware of their fall risk score higher on functional tests and engage in more physical activity, which may account for no association between AKP and recurrent falls in participants aged < 65 years in this study. Younger individuals may be more conscious of their fall risk than those ≥ 65 years of age, ultimately reducing their likelihood of falling frequently. An association was observed between AKP and recurrent falls in male participants but not in female participants in this study. The effect of pain on muscle strength may differ between males and females, and may contribute to these differences. Although data suggest that males typically have stronger quadriceps femoris muscles than females [44], it is unclear if pain affects muscle strength differently in males and females.

This study is a retrospective cohort study. In contrast to previous studies, this study focused on assessing knee pain within a specific area, as determined through examination by an experienced physician, and a sensitivity analysis was conducted to eliminate the effect of the knee joint replacement, as previous studies have reported that patients frequently develop AKP after knee joint replacement [45, 46]. Attention should be directed to certain limitations in the current research. Due to its retrospective design, the participants’ recollection of falls may have been subject to bias. Additionally, the majority of falls could have been either underreported or overreported, which could potentially impact the outcomes of the investigation. The data utilized in this study is solely sourced from the OAI database, encompassing patients who are either affected by knee OA or are at an increased risk of developing it. This exclusive reliance on the OAI database may impede the generalizability of the study findings. While acknowledging the potential for reverse confounding inherent in observational studies, we took measures to control for as many covariates as possible. It is important to note that AKP can be influenced not only by knee joint pathology, but also may by abnormal hip joint activity and changes in lower limb alignment, ultimately impacting the loading of the patellofemoral joint and leading to AKP. Further research should explore the association and severity of AKP with abnormal lower limb movement in affected individuals.

## Conclusion

In conclusion, this study demonstrates that AKP can be used to predict falls in individuals with knee OA or at high-risk of developing knee OA. Individuals with AKP are at risk for recurrent falls, especially those who are older men. Combined with the existing research on AKP and knee OA, the results of this study indicate that it is crucial to prioritize fall prevention efforts for patients with AKP instead of waiting for the progression of knee OA before initiating fall prevention measures. The rationale behind this approach lies in the possibility that the occurrence of AKP may precede the progression of knee OA.

### Electronic supplementary material

Below is the link to the electronic supplementary material.

**Supplementary Table 1**. Hierarchical analysis by age in the recurrent falls group. **Supplementary Table 2**. Hierarchical analysis by gender in the recurrent falls group. **Supplementary Table 3**. Odds Ratio (95% Confidence Intervals) for any fall in different depression group (sensitivity analyses). **Supplementary Table 4**. Odds Ratio (95% Confidence Intervals) for AKP in the recurrent falls group (sensitivity analyses).

## Data Availability

The data that support the findings of this study are available from the National Institutes of Health repository but restrictions apply to the availability of these data, which were used under license for the current study, and so are not publicly available. Data are however available from the author TX (e-mail: yzx_20190705@163.com) upon reasonable request and with permission of the National Institutes of Health repository https://nda.nih.gov/oai/.

## References

[1] Sterling DA, O’Connor JA, Bonadies J (2001). Geriatric falls: injury severity is high and disproportionate to mechanism. J Trauma.

[2] Centers for Disease Control and Prevention, National Center for Injury Prevention and Control. Web-based injury statistics query and reporting system. https://www.cdc.gov/injury/wisqars/index.html. Accessed 5 Aug 2016.

[3] Gill TM, Murphy TE, Gahbauer EA (2013). Association of injurious falls with disability outcomes and nursing home admissions in community-living older persons. Am J Epidemiol.

[4] Davis JC, Robertson MC, Ashe MC (2010). International comparison of cost of falls in older adults living in the community: a systematic review. Osteoporos International: J Established as Result Cooperation between Eur Foundation Osteoporos Natl Osteoporos Foundation USA.

[5] Burns E, Kakara R (2018). Deaths from Falls among persons aged ≥ 65 years - United States, 2007–2016. MMWR Morbidity and Mortality Weekly Report.

[6] Bergen G, Stevens MR, Burns ER (2016). Falls and fall injuries among adults aged ≥ 65 years - United States, 2014. MMWR Morbidity and Mortality Weekly Report.

[7] Leveille SG, Jones RN, Kiely DK (2009). Chronic musculoskeletal pain and the occurrence of falls in an older population. JAMA.

[8] Li Y, Liu M, Sun X (2020). Independent and synergistic effects of pain, insomnia, and depression on falls among older adults: a longitudinal study. BMC Geriatr.

[9] Hirase T, Okubo Y, Sturnieks DL (2020). Pain is Associated with Poor Balance in Community-Dwelling older adults: a systematic review and Meta-analysis. J Am Med Dir Assoc.

[10] Neogi T (2013). The epidemiology and impact of pain in osteoarthritis. Osteoarthritis Cartilage.

[11] Prevalence of disabilities and associated health conditions among adults–United States., 1999. MMWR Morbidity and mortality weekly report 2001;50(7):120–125. 11393491

[12] Powers CM, Bolgla LA, Callaghan MJ (2012). Patellofemoral pain: proximal, distal, and local factors, 2nd International Research Retreat. J Orthop Sports Phys Ther.

[13] Macri EM, Neogi T, Jarraya M (2022). Magnetic resonance imaging-defined osteoarthritis features and anterior knee Pain in individuals with, or at risk for, knee osteoarthritis: a Multicenter Study on Osteoarthritis. Arthritis Care Res (Hoboken).

[14] Stefanik JJ, Guermazi A, Roemer FW (2016). Changes in patellofemoral and tibiofemoral joint cartilage damage and bone marrow lesions over 7 years: the Multicenter Osteoarthritis Study. Osteoarthritis Cartilage.

[15] Lankhorst NE, Damen J, Oei EH (2017). Incidence, prevalence, natural course and prognosis of patellofemoral osteoarthritis: the Cohort hip and cohort knee study. Osteoarthritis Cartilage.

[16] Michael CN, FelsonTF DT, Gayle Lester M. P. The osteoarthritis initiative. https://nda.nih.gov/static/docs/StudyDesignProtocolAndAppendices.pdf. Accessed 7 Sept 2023.

[17] Michael CN, FelsonTF DT, Gayle Lester M. P. The osteoarthritis initiative-operations manuals. https://nda.nih.gov/oai/study_documentation.html. Accessed 7 Sept 2023.

[18] Munch T, Harrison SL, Barrett-Connor E (2015). Pain and falls and fractures in community-dwelling older men. Age Ageing.

[19] Deandrea S, Lucenteforte E, Bravi F (2010). Risk factors for falls in community-dwelling older people: a systematic review and meta-analysis. Epidemiol (Cambridge Mass).

[20] Katz JN, Chang LC, Sangha O (1996). Can comorbidity be measured by questionnaire rather than medical record review?. Med Care.

[21] Cosco TD, Lachance CC, Blodgett JM (2020). Latent structure of the centre for epidemiologic studies Depression Scale (CES-D) in older adult populations: a systematic review. Aging Ment Health.

[22] Washburn RA, Smith KW, Jette AM (1993). The physical activity scale for the Elderly (PASE): development and evaluation. J Clin Epidemiol.

[23] Michael CN, FelsonTF DT, Gayle Lester M. P. The osteoarthritis initiative-study design and general analysis information. https://nda.nih.gov/oai/study_documentation.html. Accessed 7 Sept 2023.

[24] Shea CA, Ward RE, Welch SA (2018). Inability to perform the repeated chair stand Task predicts fall-related Injury in Older Primary Care patients. Am J Phys Med Rehabil.

[25] Kwon SC, Han BH, Kranick JA (2018). Racial and ethnic difference in Falls among older adults: results from the California health interview survey. J Racial Ethnic Health Disparities.

[26] Centers for Disease Control and Prevention/Body Mass Index. (BMI). Healthy weight, nutrition, and physical activity. https://www.cdc.gov/healthyweight/assessing/index.html. Accessed 7 Sept 2023.

[27] Riddle DL, Golladay GJ (2016). A longitudinal comparative study of falls in persons with knee arthroplasty and persons with or at high risk for knee osteoarthritis. Age Ageing.

[28] Studenski S, Perera S, Patel K (2011). Gait speed and survival in older adults. JAMA.

[29] Sharma L, Chmiel JS, Almagor O (2015). Knee instability and basic and advanced function decline in knee osteoarthritis. Arthritis Care Res (Hoboken).

[30] Lewinsohn PM, Seeley JR, Roberts RE (1997). Center for epidemiologic studies Depression Scale (CES-D) as a screening instrument for depression among community-residing older adults. Psychol Aging.

[31] Hoffman GJ, Ha J, Alexander NB (2018). Underreporting of fall injuries of older adults: implications for Wellness visit fall risk screening. J Am Geriatr Soc.

[32] Lo-Ciganic WH, Floden L, Lee JK (2017). Analgesic use and risk of recurrent falls in participants with or at risk of knee osteoarthritis: data from the Osteoarthritis Initiative. Osteoarthritis Cartilage.

[33] Harris R, Strotmeyer ES, Sharma L (2023). The Association between Severity of Radiographic Knee OA and recurrent falls in Middle and older aged adults: the Osteoarthritis Initiative. The Journals of Gerontology Series A Biological Sciences and Medical Sciences.

[34] Jehu DA, Davis JC, Falck RS (2021). Risk factors for recurrent falls in older adults: a systematic review with meta-analysis. Maturitas.

[35] Arden NK, Crozier S, Smith H (2006). Knee pain, knee osteoarthritis, and the risk of fracture. Arthritis Rheum.

[36] Muraki S, Akune T, Oka H (2011). Prevalence of falls and the association with knee osteoarthritis and lumbar spondylosis as well as knee and lower back pain in Japanese men and women. Arthritis Care Res (Hoboken).

[37] Patel KV, Phelan EA, Leveille SG (2014). High prevalence of falls, fear of falling, and impaired balance in older adults with pain in the United States: findings from the 2011 National Health and Aging trends Study. J Am Geriatr Soc.

[38] Hicks C, Levinger P, Menant JC (2020). Reduced strength, poor balance and concern about falls mediate the relationship between knee pain and fall risk in older people. BMC Geriatr.

[39] Werner S (2014). Anterior knee pain: an update of physical therapy. Knee Surgery, sports traumatology, arthroscopy. Official J ESSKA.

[40] Cibulka MT, Threlkeld-Watkins J (2005). Patellofemoral pain and asymmetrical hip rotation. Phys Ther.

[41] Souza RB, Powers CM (2009). Differences in hip kinematics, muscle strength, and muscle activation between subjects with and without patellofemoral pain. J Orthop Sports Phys Ther.

[42] Assa T, Elbaz A, Mor A (2013). Gait metric profile of 157 patients suffering from anterior knee pain. A controlled study. Knee.

[43] Moreira NB, Rodacki ALF, Pereira G (2018). Does functional capacity, fall risk awareness and physical activity level predict falls in older adults in different age groups?. Arch Gerontol Geriatr.

[44] Sinaki M, Nwaogwugwu NC, Phillips BE (2001). Effect of gender, age, and anthropometry on axial and appendicular muscle strength. Am J Phys Med Rehabil.

[45] van Jonbergen HP, Reuver JM, Mutsaerts EL (2014). Determinants of anterior knee pain following total knee replacement: a systematic review. Knee Surgery, sports traumatology, arthroscopy. Official J ESSKA.

[46] Petersen W, Rembitzki IV, Brüggemann GP (2014). Anterior knee pain after total knee arthroplasty: a narrative review. Int Orthop.

