# Supplementary material for: Anterior knee pain as a potential risk factor for falls in older adults: insights from the osteoarthritis initiative data
Source: BMC Public Health. 2023 Nov 20;23:2288. doi: 10.1186/s12889-023-17237-8 (PMC10662569; doi:10.1186/s12889-023-17237-8)
Supplement: Supplementary file 1 — Supplementary Table 1. Hierarchical analysis by age in the recurrent falls group. Supplementary Table 2. Hierarchical analysis by gender in the recurrent falls group. Supplementary Table 3. Odds Ratio (95% Confidence Intervals) for any fall in different depression group (sensitivity analyses). Supplementary Table 4. Odds Ratio (95% Confidence Intervals) for AKP in the recurrent falls group (sensitivity analyses). [file 12889_2023_17237_MOESM1_ESM.docx]

**Supplementary Table 1. Hierarchical analysis by age in the recurrent falls group.**

|  | **Age<65** | | | **Age≥65** | | |
| --- | --- | --- | --- | --- | --- | --- |
|  | OR | 95%CI | P | OR | 95%CI | P |
| AKP *(reference of non-AKP)* |  |  |  |  |  |  |
| Unadjusted Model | 1.50 | 1.23-1.83 | <0.001 | 1.81 | 1.38-2.37 | <0.001 |
| Multivariate Model^†^ | 1.19 | 0.95-1.50 | 0.126 | 1.40 | 1.06-1.87 | 0.019 |

^†^Age, BMI, gender, race, education, cohort, history of smoking, alcohol use, analgesics use, history of fall, Charlson comorbidity index, CES-D, PASE, Repeated chair stands, pace, and knee confidence.

**Supplementary Table 2. Hierarchical analysis by gender in the recurrent falls group.**

|  | **Female** | | | **Male** | | |
| --- | --- | --- | --- | --- | --- | --- |
|  | OR | 95%CI | P | OR | 95%CI | P |
| AKP *(reference of non-AKP)* |  |  |  |  |  |  |
| Unadjusted Model | 1.49 | 1.22-1.82 | <0.001 | 1.90 | 1.44-2.50 | <0.001 |
| Multivariate Model^†^ | 1.17 | 0.94-1.45 | 0.161 | 1.45 | 1.07-1.96 | 0.017 |

^†^Age, BMI, gender, race, education, cohort, history of smoking, alcohol use, analgesics use, history of fall, Charlson comorbidity index, CES-D, PASE, Repeated chair stands, pace, and knee confidence.

**Supplementary Table 3. Odds Ratio (95% Confidence Intervals) for any fall in different depression group *(sensitivity analyses).***

|  | **CES-D<16** | | | **CES-D≥16** | | |
| --- | --- | --- | --- | --- | --- | --- |
|  | OR | 95%CI | P | OR | 95%CI | P |
| AKP *(reference of non-AKP)* |  |  |  |  |  |  |
| Unadjusted Model | 1.38 | 1.20-1.58 | <0.001 | 1.00 | 0.72-1.39 | 0.997 |
| Multivariate ModeL^†^ | 1.22 | 1.06-1.41 | 0.006 | 0.88 | 0.62-1.25 | 0.454 |

^†^Age, BMI, gender, race, education, cohort, history of smoking, alcohol use, analgesics use, history of fall, Charlson comorbidity index, PASE, Repeated chair stands, pace, and knee confidence. NOTE: CES-D × AKP interaction, P =0.011

**Supplementary Table 4. Odds Ratio (95% Confidence Intervals) for AKP in the recurrent falls group *(sensitivity analyses).***

|  | **Recurrent Falls** | | |
| --- | --- | --- | --- |
|  | OR | 95%CI | P |
| AKP *(reference of non-AKP)* |  |  |  |
| Unadjusted model | 1.59 | 1.33-1.88 | <0.001 |
| Multivariate and physical function adjusted mode^†^ | 1.26 | 1.04-1.52 | 0.020 |

^†^Age, BMI, gender, race, education, cohort, history of smoking, alcohol use, analgesics use, history of fall, Charlson comorbidity index, CES-D, PASE, Repeated chair stands, pace, and knee confidence. NOTE: CES-D × AKP interaction, P =0.051.
